# Insights Into the Complexity of Yeast Extract Peptides and Their Utilization by *Streptococcus thermophilus*

**DOI:** 10.3389/fmicb.2019.00906

**Published:** 2019-04-30

**Authors:** Lucas Proust, Alain Sourabié, Martin Pedersen, Iris Besançon, Eloi Haudebourg, Véronique Monnet, Vincent Juillard

**Affiliations:** ^1^Micalis Institute, INRA, AgroParisTech, Université Paris-Saclay, Jouy-en-Josas, France; ^2^Procelys, Lesaffre Group, Maisons-Alfort, France; ^3^Sacco S.r.l., Cadorago, Italy

**Keywords:** *Streptococcus thermophilus*, yeast extract, peptidomics, oligopeptide, transport

## Abstract

*Streptococcus thermophilus*, an extensively used lactic starter, is generally produced in yeast extract-based media containing a complex mixture of peptides whose exact composition remains elusive. In this work, we aimed at investigating the peptide content of a commercial yeast extract (YE) and identifying dynamics of peptide utilization during the growth of the industrial *S. thermophilus* N4L strain, cultivated in 1 l bioreactors under pH-regulation. To reach that goal, we set up a complete analytical workflow based on mass spectrometry (peptidomics). About 4,600 different oligopeptides ranging from 6 to more than 30 amino acids in length were identified during the time-course of the experiment. Due to the low spectral abundance of individual peptides, we performed a clustering approach to decipher the rules of peptide utilization during fermentation. The physicochemical characteristics of consumed peptides perfectly matched the known affinities of the oligopeptide transport system of *S. thermophilus*. Moreover, by analyzing such a large number of peptides, we were able to establish that peptide net charge is the major factor for oligopeptide transport in *S. thermophilus* N4L.

## Introduction

Lactic acid bacteria (LAB) are a group of microorganisms displaying a wide range of properties making them suitable for various applications in fields such as health (Hill et al., 2017), chemistry (Othman et al., 2017; Sauer et al., 2017), or cosmetics (Izawa and Sone, 2014). However, they have been historically used for food production (Salque et al., 2013), and it still remains their main outcome, in particular for dairy starters. Therefore, detailed information is available about the growth of LAB in milk, especially regarding the proteolytic system responsible for their amino nitrogen nutrition (Kunji et al., 1996; Christensen et al., 1999). When considering the whole lifetime of dairy starters, the culture medium in which they are industrially produced also represents an important substrate for these bacteria. In contrast to milk culture, less information has been published on this particular step, although it is of great importance as it impacts both production yields and technological functionalities of the starter (Ummadi and Curic-Bawden, 2008). Production media usually contain complex substrates such as cell or protein hydrolysates that notably include yeast extracts (YEs), which are widely used for LAB industrial production. YEs correspond to the soluble fraction of molecules released after either yeast autolysis or controlled enzymatic lysis (Pasupuleti and Braun, 2008). They contain several classes of nutrients among which peptides that can represent more than 50% of the total YE mass, depending on the manufacturing process. They are thus mainly employed as amino nitrogen sources. If the importance of peptides in milk cultures has been extensively covered – especially with the LAB model *Lactococcus lactis* (Juillard et al., 1995, 1998; Kunji et al., 1995; Helinck et al., 2003) – less is known about their utilization in YE-based media.

On one hand, peptide utilization in YE naturally depends on the composition of the peptide fraction, which precisely constitutes the main hurdle to this study due to the abundance and diversity of peptides composing these substrates. To the best of our knowledge, the YE peptide fraction has only been characterized up to now *via* filtration or chromatographic fractionation strategies (Mosser et al., 2011, 2015; Spearman et al., 2014, 2016), or by analyzing the composition in peptide-bound amino acids (Kevvai et al., 2014, 2016). Nonetheless, the exact nature of the peptides composing YEs still remains elusive, these compounds are therefore commonly considered as black boxes containing a complex mixture of undefined peptides.

On the other hand, peptide utilization is also dependent on the transport machinery of the cultivated LAB. Indeed, the only known LAB peptidases able to convert peptides into free amino acids are intracellularly located (Christensen et al., 1999; Savijoki et al., 2006). Several peptide transport systems are available in LAB among which two are particularly well documented and present in various species. The first one is the di- and tri-peptide transporter DtpT, a secondary transporter belonging to the PTR (peptide transport) family. It is a protein encoded by a single gene and constituted of 12 transmembrane domains that couples peptide transport with the proton gradient (Hagting et al., 1997). It has been identified in several LAB, among which *L. lactis* (Hagting et al., 1994), *Lactobacillus helveticus* (Nakajima et al., 1997), or *Streptococcus thermophilus* (Hols et al., 2005; Goh et al., 2011). The second well-known system for peptide uptake is an ABC (ATP-binding cassette) transporter dedicated to oligopeptides (3 residues and more). It is known as Opp in many LAB species, in particular in *L. lactis* where it has first been characterized (Tynkkynen et al., 1993). It has also been found in *S. thermophilus* where it has been named Ami due to its high sequence homology with other streptococcal transporters (Garault et al., 2002). Both Opp and Ami share a similar overall genetic organization. They consist of five conserved proteins organized in a single operon: OppDFBCA and AmiACDEF, respectively. OppA and AmiA are lipoproteins anchored to the cell membrane and devoted to peptide binding and delivery to the translocon formed by OppBC/AmiCD. Peptide internalization is enabled by the cytoplasmic membrane-bound ATPases OppDF/AmiEF that energize the whole system upon ATP binding and hydrolysis. However, the Ami system possesses supplementary AmiA proteins, in opposition to OppA present in only one copy, which is a characteristics of streptococci. Extra AmiA proteins are located in other parts of the genome, and their number is strain-dependent.

These two combined systems, DtpT and Opp/Ami, allow for a large supply of various peptides to the bacterial cells. However, peptide length is not the sole factor upon which these carriers operate, as peptides are not indiscriminately transported even when belonging to the adequate size range. In the case of DtpT, it has been evidenced in several species that it had a higher affinity for dipeptides over tripeptides, preferred hydrophobic substrates and worked less efficiently with cationic peptides (Nakajima et al., 1997; Fang et al., 2000; Solcan et al., 2012; Martinez Molledo et al., 2018). Concerning the oligopeptide carrier specificity, extensive information is available about *L. lactis* Opp. Specifically, *in vivo*, *in vitro*, and structural characterization data are available (Tynkkynen et al., 1993; Juillard et al., 1995, 1998; Detmers et al., 1998, 2000; Kunji et al., 1998; Lanfermeijer et al., 2000; Charbonnel et al., 2003; Helinck et al., 2003; Doeven et al., 2004, 2005; Berntsson et al., 2009, 2011). In comparison, apart from its initial genetic identification and characterization (Garault et al., 2002), only one *in vivo* study has been performed on *S. thermophilus* Ami transporter to determine its substrate preferences (Juille et al., 2005). Complementary approaches based on the use of mixtures of milk peptides showed that transport seemed to be in favor of hydrophobic and positively charged oligopeptides, whereas long anionic ones were never taken up. However, this study was based on a limited number of peptides notably resulting from the tryptic digestion of α_s2_-casein and therefore presenting biochemical similarities such as a positively charged C-terminal residue (Lys or Arg). One can therefore question whether the trends shown by the consumption of such limited and specific peptide mixtures are representative of the Ami oligopeptide transporter specificities, and furthermore whether they can be extended to a YE-based medium where oligopeptides are abundant and available in the form of a large complex mix.

In this study, we specifically aimed at qualitatively characterizing the oligopeptide fraction of a YE-based growth medium and monitoring the dynamics of oligopeptide utilization occurring during the growth of an industrial *S. thermophilus* strain. For that purpose, we developed a specific peptidomics-based analytical pipeline combined with a dedicated data analysis workflow. This whole approach revealed the complexity of the YE peptide fractions as well as peptide utilization dynamics that notably reflected the activity and the specificity of the oligopeptide transporter Ami.

## Materials and Methods

### Strain and Preculture Conditions

This work used *S. thermophilus* N4L (Proust et al., 2018), a PrtS-positive, AmiA2-negative and AmiA3-positive industrial starter. This strain also contains the gene encoding the DtpT transporter, and does not possess the Ots peptide transport system present in some other *S. thermophilus* strains (Goh et al., 2011; Jameh et al., 2016). Therefore, DtpT and Ami are the only known peptide transport systems identified in this strain. It was stored at -80°C in M17 broth (Terzaghi and Sandine, 1975) containing 1% (w/v) lactose and supplemented with 20% (v/v) glycerol. The strain was routinely pre-cultured in M17 broth containing 5% lactose at 42°C.

### Bioreactor Culture Conditions

Bioreactor cultures were performed in a 1 l bioreactor system BIOSTAT^®^ Qplus (Sartorius Stedim Biotech, Germany). Two successive precultures were performed prior to inoculating at 2% (v/v) the fermenters. The culture medium contained (w/v) 6% lactose, 0.01% calcium chloride and 2% of YE provided by Procelys (Maisons-Alfort, France) belonging to the Nucell^®^ range notably dedicated to dairy starters. A pool of vitamins as used in *S. thermophilus* chemically defined medium (Letort and Juillard, 2001) was also added in the culture medium in order to ensure repeatable and optimal growth performances similar to those obtained with equivalent Tween 80-containing media (data not shown). Tween 80 is a growth factor widely used for LAB cultures (Williams et al., 1947). However, it is a highly ionizable compound known to disrupt mass spectrometry analyses (Jäpelt et al., 2016) and thus was not useable in this study. The YE fraction of the medium was sterilized by heat treatment along with the bioreactors for 20 min at 120°C. The other components were sterilized by a 0.22 μm pore-sized polyethersulfone membrane (Millipore, Guyancourt, France). The initial pH was adjusted beforehand to 6.6 with sodium hydroxide 2 M. The batch fermentations were carried out during 6 h at 40°C, the stirring was fixed at 50 rpm and the pH regulated at 6.0 with sodium hydroxide 2 M. Pseudo anaerobic conditions were set up by sparging nitrogen at 0.2 l/min in the growth medium for 1 h before inoculation, and in the headspace at the same flow rate thereafter. Growth was followed by optical density (600 nm) measurement and by online monitoring of the volume of base added.

Three independent cultures were carried out during which samples were collected each hour for the first repetition. In order to maximize reproducibility, samples from the two subsequent repetitions were taken when the added volumes of base reached the corresponding levels of the first repetition.

### Yeast Peptide Identification

**FIGURE 1:**
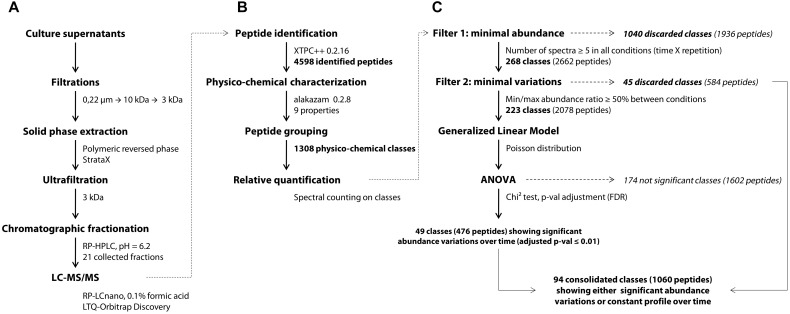
General workflow of the peptidomics analytical pipeline, starting from the experimental part **(A)** to the bioinformatic **(B)** and statistical analyses **(C)**. XTPC++, X!Tandem Pipeline (C++).

YE peptides in the culture medium were identified using an approach adapted from a previous work (Guillot et al., 2016). Bacterial cells were first discarded from the fermentation samples by centrifugation (3000 g, 10 min, 4°C). The peptide-containing supernatants were filtered using a 0.22 μm pore-sized PVDF membrane with low protein binding properties (Millipore). Trifluoroacetic acid (TFA) and acetonitrile (ACN) were then added at final concentrations of, respectively, 0.1 and 5% (v/v). The mixes were centrifuged (3000 g, 10 min, 4°C) after a one-night storage at 4°C, then ultrafiltered successively through 10 and 3 kDa cut-off Amicon^®^ Ultra-15 devices (Ultracel^®^-10K and Ultracel^®^-3K membranes, resp., Millipore). The final permeates went through a solid phase extraction step using a 200 mg StrataX^®^ cartridge according to manufacturer procedure (Phenomenex, Le Pecq, France). Briefly, the activated cartridge was loaded with 4 ml of sample, washed with 5% ACN and 0.1% TFA, and peptides were finally eluted with 1.5 ml of 50% ACN and 0.1% TFA in MilliQ water (Waters, St-Quentin-en-Yvelines, France). The eluted fractions obtained were then dried overnight in a Speed-Vac system (Savant, Thermo Fisher Scientific France, Illkirch, France) and resolubilized in 400 μl of 0.1% TFA. Finally, the concentrates were ultrafiltered once again through a 3 kDa cut-off Amicon^®^ Ultra-4 device to remove potential insoluble materials which might clogg column and spectrometer prior to a double chromatographic separation (Figure 1A).

The first separation was performed on a Nucleoshell RP 18plus reversed-phase column (150 × 4.6 mm, 2.7 μm, 87.5 Å, Macherey-Nagel, Germany) at 40°C with an injection volume of 25 μl corresponding to 250 μl of the initial supernatant. A linear gradient of acetonitrile (1.6% per min) in ammonium formate (20 mM, pH 6.2) was applied with a 0.7 ml per min flow rate and fractions were collected every minute. Preliminary analyses showed that the initial and last fractions collected, respectively, before 5 min and after 25 min of the chromatographic run contained less than 5% of the identified peptides (data not shown) and thus were discarded. The remaining 21 fractions were subsequently dried overnight in a SpeedVac system and resuspended in 30 μl of 0.1% TFA and 2% ACN in MilliQ water. Aliquots of 5 μl were analyzed by a data-dependent tandem mass spectrometry approach on the PAPPSO platform (INRA, Jouy-en-Josas). All the *modus operandi* of the second chromatographic separation and peptide m/z detection were the same as those previously described (Guillot et al., 2016). Peptides were separated on a Pepmap C18 column (150 mm × 0.75 mm) at 300 nl/min with a gradient of ACN in formic acid. Eluted peptides were analyzed online on an LTQ-Orbitrap Discovery mass spectrometer (Thermo Fisher Scientific). Peptide ionization was performed with a spray voltage of 1.3 kV. Peptide ions were analyzed by the data-dependent method as follows: full MS scan (m/z 350–1,600) was performed on the Orbitrap mass analyzer and the six most abundant doubly and triply charged peptides were submitted to MS/MS analysis with a collision energy of 35%. An exclusion window of 40 s was applied. Peptide identification was performed with X!Tandem version 2015.12.15.2 (Vengeance) and X!TandemPipeline (C++) version 0.2.16 (Langella et al., 2016) on the protein sequence of *Saccharomyces cerevisiae* S288C (version 2015-01-13) ^1^. The main peptide identification parameters were the following: no cleavage specificity, variable methionine oxidation state and mass tolerance for parent and fragment ions of ± 10 ppm and ± 0.4 Da, respectively. Peptides were conserved when showing an *E*-value ≤ 0.05, and only one peptide per parental protein was considered as sufficient to enable identification. Contaminant peptides were discarded using a standard proteomic contaminant database, and the False Discovery Rate was estimated using the reversed protein database.

### Peptide Physicochemical Characterization and Class Assignment

Peptides were characterized by nine different physicochemical properties listed in Table 1. At the exception of the proportions of aromatic residues and proline which were manually calculated, all other properties were computed using the aminoAcidProperties function of the R package “alakazam” version 0.2.8 (Gupta et al., 2015). Default settings were kept for scaling and normalization procedures.

**Table 1:** Peptide physicochemical properties and interval division used for peptide barcoding.

Peptide property	Details	Interval 1	Interval 2	Interval 3
Length	Number of amino acids	≤9	[10; 12]	≥13
GRAVY	Grand average of hydropathy index (Kyte and Doolittle, 1982)	≤–2	]-2; -0.5]	>–0.5
Bulkiness	Average section of amino acid side chains, in Å^2^ (Zimmerman et al., 1968)	≤12.6	]12.6; 14.7]	>14.7
Polarity	Average polarity of amino acids (Grantham, 1974)	≤8.7	]8.7; 10.1]	>10.1
Net charge	Normalized net charge calculated at pH = 6.0 (Moore, 1985)	<0	[0;1]	>1
Basic residues	Proportion of lysine, arginine and histidine	=0	]0; 0. 12]	>0. 12
Acidic residues	Proportion of aspartic and glutamic acids	=0	]0; 0. 12]	>0. 12
Aromatic residues	Proportion of phenylalanine, tryptophan and tyrosine	=0	]0; 0. 12]	>0. 12
Proline	Proportion of proline	=0	]0; 0. 12]	>0. 12


**FIGURE 2:**
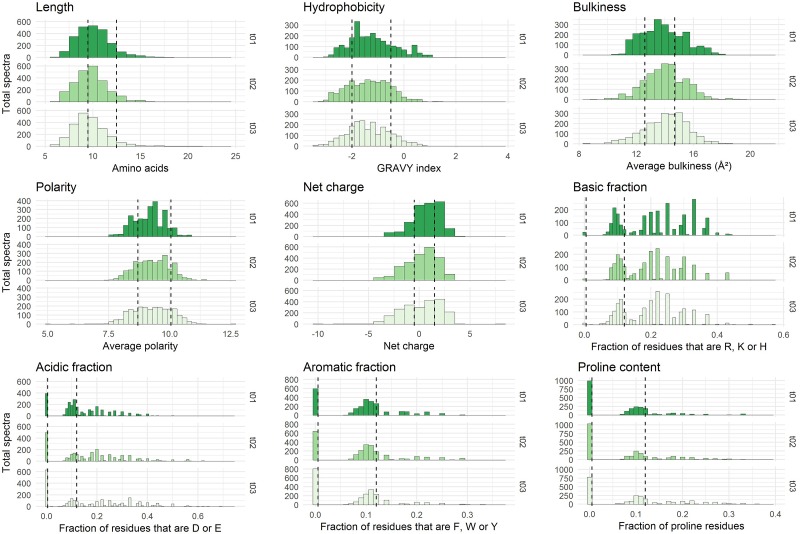
Physicochemical properties of the peptides initially present in YE before inoculation (*t* = 0 h). The three repetitions are represented separately. The x-axes correspond to the scales specific to each physicochemical property. The y-axes represent the number of spectra observed. Vertical dashed lines indicate the limits chosen for the definition of the intervals used for peptide barcoding (Table 1). As peptide length, hydrophobicity, bulkiness, polarity, and net charge show a bell-shaped distribution, the limits were chosen so that they framed the profile peak. The remaining properties correspond to frequencies of specific types of residues in the peptide amino acid composition. The first limit defines the “zero sub-class”: absence of the considered residues in the peptide composition, and the second limit was arbitrarily fixed at 12%.

Prior to differential analysis, peptides were grouped together in classes based on their physicochemical closeness (Figure 1B). For that purpose, a specific procedure was performed in order to assign every identified peptide to a unique physicochemical class. The range of each descriptor was divided into three intervals (Table 1 and Figure 2). For each physicochemical criterion, a given peptide can belong to only one interval. The physicochemical class or “barcode” of the peptide is then defined as the combination of the respective intervals of the nine descriptors. As an example, the peptide KGSIDEQHPRYGG belongs to the class “321223322.” It is 13-residues long, therefore it belongs to the interval 3 of length, its GRAVY index value is -1.73: interval 2, and so on.

### Statistical Analyses

Wilcoxon–Mann–Whitney (Wilcoxon, 1945; Mann and Whitney, 1947) and Kruskal–Wallis (Kruskal and Wallis, 1952) non-parametric tests were used in order to detect statistical differences in peptide property distributions between 2 or more than 2 groups, respectively. For that purpose, the *ad hoc* functions of the R package “stats” version 3.4.3 were employed.

Differential analysis over time was performed on the abundance of peptide classes with the R script MassChroqR (version 0.3.8) of the MassChroQ pipeline (Valot et al., 2011). A contingency table was generated beforehand that contained the total spectral counts of each physicochemical class – i.e., the sum of the spectral counts of their constitutive peptides – in each analyzed sample. Classes showing low abundance (<5 spectra in all samples) and little variations (less than 50% of variation between the minimal and maximal average abundance observed in the different samples) were discarded. Finally, spectral count data of remaining classes were modeled *via* a generalized linear model with a Poisson distribution. An analysis of variance (ANOVA) was then performed using a Chi^2^ test to detect significant variations in peptide class abundances with time considered as factor of analysis. The generated *p*-values were adjusted by a FDR procedure (Benjamini and Hochberg, 1995). Classes showing an adjusted *p* ≤ 0.01 were considered as varying significantly over time (Figure 1C). A kinetic profiling was then manually performed in order to separate classes showing either a decrease, increase or fluctuating variations over-time. The physicochemical properties of peptides belonging to the different kinetic profiles were then discriminated by a principal component analysis (PCA) (R package “FactoMineR” version 1.41, Lê et al., 2008).

## Results

*S. thermophilus* N4L was cultivated in a YE-based medium in 1 l bioreactors. The peptide content of the culture supernatants was monitored during growth using mass spectrometry. Peptide identification was performed on the initial medium before inoculation (*t* = 0 h) and then each hour from 3 to 6 h of growth, corresponding to the exponential and the early stationary growth phases (Supplementary Figure S1).

### YE Contains a Large Number of Peptides With Different Levels of Abundance

Between 1,300 and 1,700 distinct peptides were identified per analyzed time point from approximately 1,900 to 2,600 fragmentation spectra, depending on the point considered (Table 2). These values were consistent within biological repetitions as indicated by the low coefficients of variation (average variation around the mean of 7% both at peptide and spectra levels), showing a good reproducibility in terms of number of identified peptides. Nevertheless, the qualitative identification of peptides was not as effective, as, for a given time, only an average of 55% of the peptides was identified in all three repetitions. This confirms that non-tryptic peptide identification in complex mixtures is still technically challenging, as already discussed (Guillot et al., 2016). Combining all the identifications from all time points and repetitions resulted in a total of 4,598 distinct peptides identified (FDR < 1%) from 32,920 fragmentation spectra during the course of the growth.

**Table 2:** Summary of peptide identification during growth of *S. thermophilus* N4L in a YE-based medium.

	*t* = 0 h	*t* = 3 h	*t* = 4 h	*t* = 5 h	*t* = 6 h
Peptides	1560 ± 69	1742 ± 63	1514 ± 123	1402 ± 124	1290 ± 107
Total spectra	2294 ± 86	2596 ± 119	2211 ± 157	2010 ± 198	1863 ± 190


*Means of 3 independent repetitions ± standard deviation.*

To estimate peptide relative abundance, spectral counting is considered as the simplest method in a label-free approach (Liu et al., 2004; Colinge et al., 2005). Table 3 shows that the majority of peptides (close to 80%) was actually scarcely identified with only one spectrum per peptide. Even though label-free mass spectrometry only allows relative quantification, these peptides are likely to be either the less abundant ones, or to have poor yields of detection. Nevertheless, some peptides generated larger numbers of spectra. In particular, the top ones (more than 10 spectra per peptide) represented less than 1% of the identified peptides in each sample but 5–10% of the total number of spectra. Thus, they are likely to be the most abundant in the medium. All these data provide evidence that this YE contains a high peptide diversity with a few of them being over-abundant.

**Table 3:** Average number of peptides identified as a function of their relative abundance.

Number of spectra per peptides	*t* = 0 h	*t* = 3 h	*t* = 4 h	*t* = 5 h	*t* = 6 h
More than 10	11 ± 0	14 ± 9	9 ± 1	11 ± 2	10 ± 4
From 5 to 9	40 ± 5	44 ± 8	37 ± 4	25 ± 5	27 ± 1
From 2 to 4	272 ± 18	316 ± 5	273 ± 22	252 ± 37	230 ± 40
1	1236 ± 55	1369 ± 60	1195 ± 102	1113 ± 86	1023 ± 67


*Means of 3 independent repetitions ± standard deviation.*

### Peptide Physicochemical Properties

Each identified peptide was characterized by a combination of nine physicochemical properties. These properties have been chosen to describe comprehensively YE peptide diversity. They are summarized in Table 1. The internal distributions of each property calculated from the initial medium peptidome (before inoculation, *t* = 0 h) are represented in Figure 2. No significant difference could be detected (*p* ≤ 0.01) between the three repetitions, and the other time points of analysis showed good reproducibility as well (Supplementary Figure S2).

Most of the nine physicochemical properties of the peptides initially identified were distributed within a relatively broad range, reflecting a large physicochemical diversity. The detected peptides were mostly hydrophilic, had a median net charge slightly positive, and their average bulkiness was close to 14 Å^2^ which is moderately inferior to that of the mean of the 20 standard amino acids, ca. 15.4 Å^2^ (Zimmerman et al., 1968). This last finding suggests a slight over-representation in the yeast-derived peptide sequences of relatively small residues. Finally, these identified peptides showed an average length of 10 residues, although this observation has to be tempered by the specificities of the analytical pipeline. Indeed, the upper length limit was driven by the purification process employed and more specifically by the 3 kDa ultrafiltration steps, while the lower limit – no detection of peptides shorter than 6 residues – was a direct consequence of the chosen mass spectrometry range detection (350–1,600 m/z).

### Identification of Peptide Kinetic Dynamics

The YE is composed of a large number of peptides displaying various physicochemical properties. This inherent diversity made it suitable to study peptide utilization dynamics during the strain growth. However, as previously described, most of the identified peptides showed intermediate to low levels of spectral abundance (Table 3), which limits the relevance of a kinetic study directly at the single peptide scale. Therefore, in order to identify which peptides were utilized and on which physicochemical basis, a specific analytical workflow was developed. The underlying idea was to pool peptides showing close physicochemical properties into groups in order to combine their spectral counts and therefore perform the study not with individual peptides but on a larger scale (see section Materials and Methods section for explanations about the grouping procedure). The limits of each interval chosen during the grouping were fixed according to the internal distribution of each physicochemical property established from the initial peptidome (*t* = 0 h) before inoculation (see Figure 2 for the representation of these intervals and the general rules regarding their constitution). Theoretically, there are 3^9^ = 19,683 different possible classes or “barcodes.” Practically, not all are physically possible or exist biologically, and experimentally only 1,308 were identified here. On this total, 612 classes (47%) contained only one peptide, whereas the top three most abundant classes pooled 41, 45 (two *ex aequo* classes) and 53 peptides, respectively.

After determining their relative abundance by summing the spectral counts of their constitutive peptides, these classes were submitted to statistical analyses (Figure 1C). A first filter was applied to remove all classes showing low levels of abundance (less than 5 spectra per class) as their quantification over time would not be relevant. At this step, 1,040 classes were discarded, i.e., 80% of the total. Then, a second filter was used on the 268 remaining abundant classes in order to remove those considered largely constant, i.e., showing less than 50% of variation between their minimal and maximal abundance values in the different samples. A total of 45 classes (3%) matched this description and were not included in the following differential analysis. An analysis of variance was finally performed on the 223 remaining abundant classes that showed sufficient variation amplitude.

A total of 49 classes were declared as varying significantly over time at the threshold of an adjusted *p* ≤ 0.01. Their kinetic profiles are depicted in Supplementary Figure S3. These profiles were classified into 3 different groups: classes showing an unambiguous decrease (36 classes) or increase (2 classes) over time, and classes whose time-course evolution was fluctuating (11 classes). However, on a bacterial physiology point of view, it is of interest to characterize not only peptides that are utilized by the strain but also those that are left aside in the external medium. On this basis, the former 45 constant classes that were discarded during the second filtering procedure were therefore reintegrated with the 49 others for the last part of the analysis. In total, 94 classes were selected and can thus be classified into four different profiles whose main characteristics are given in Table 4. They enclosed a total of 1,060 different peptides, which represents about 23% of the total identified peptides (4,598), but enclosed about one third (10,412) of the total spectra (32,920) assigned during the whole experiment. The remaining classes corresponded to the combination of low abundance classes and abundant ones whose variations were not detected as significant.

**Table 4:** Characteristics of the kinetic profiles.

	Decreasing	Increasing	Constant	Fluctuating
Number of classes	36	2	45	11
Total number of peptides	342	50	584	84
Total number of spectra	2785	617	6374	636


### Linking Kinetic Profiles to Peptide Composition

**FIGURE 3:**
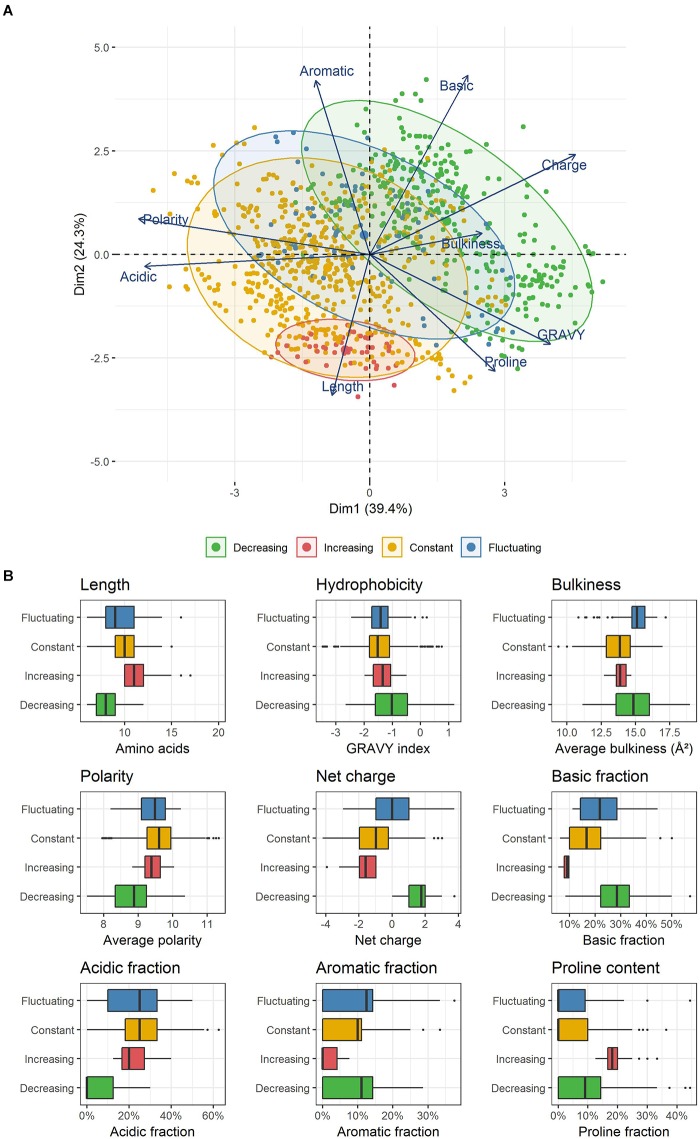
**(A)** PCA representation of peptides enclosed within the different kinetic profiles observed. Each point represents a unique peptide. **(B)** Physicochemical properties of the peptides constituting the four kinetic profiles identified. Each box plot displays the distribution of the values taken by all unique peptides included in each kinetic profile. The boxes display the range between the first and third quartile of each distribution, and the central bold lines their median value. Individual points are peptides considered as outliers.

In an attempt to correlate those different kinetic dynamics with the physicochemical properties used to design the former selected classes, a PCA was performed (Figure 3A). The two first components explained more than 60% of the total inertia. With the exception of peptide bulkiness and proline content, all other peptide properties were well represented with correlation coefficients > |0.6| on these axes. In order of importance, the first axis separated the peptides according to their polarity, acidic content, charge, hydrophobicity and, to a lesser extent, peptide bulkiness. The second axis mostly wore the information of the basic and aromatic content as well as peptide length. Proline frequency was equally supported by both axes. This representation allowed the segregation of the four kinetic profiles previously identified (increasing, decreasing, constant, fluctuating).

Peptides belonging to both constant and fluctuating profiles were spread in wide and partially overlapping areas, reflecting a common high physicochemical diversity. In contrast, decreasing and increasing profiles were located in two specific and distinct zones. Strikingly, decreasing profile corresponded to exclusively positively charged peptides (Figure 3B) that were also significantly shorter than average (*p* ≤ 0.01, median length of 8 amino acids). The positive charge was the result of both a higher and/or lower proportion of basic and acidic residues than other peptides, respectively. Moreover, these peptides were less polar, and they also contained a higher proportion of hydrophobic residues. Their aromatic and proline content was more variable and did not seem to constitute a relevant discriminative factor. In contrast, increasing profile was made up of exclusively negatively charged peptides that were also significantly longer (median length of 11 amino acids) and contained a higher amount of proline (median content = 18%). Finally, peptides enclosed in constant and fluctuating profiles displayed intermediate distributions regarding their length and net charge. Their overall hydrophobicity was not significantly different to that of increasing profile, and both displayed low and similar proline content. As an illustration, the most abundant peptides belonging to each of these kinetic profiles as well as their spectral abundance evolution are given in Table 5.

**Table 5:** Spectral abundance of the most abundant peptides enclosed within the kinetic profiles.

Profile	Peptide	Class	*t* = 0 h	*t* = 3 h	*t* = 4 h	*t* = 5 h	*t* = 6 h
Decreasing	GTWQRPH	112233133	8 ± 3	4 ± 2	2 ± 1	0 ± 1	0 ± 0
	KDIPVPKPK	123233213	11 ± 1	9 ± 2	8 ± 3	1 ± 1	1 ± 0
	GFGRIGR	131133131	11 ± 2	4 ± 1	3 ± 3	3 ± 1	4 ± 1
	RDEDKSKWMGK	212333321	8 ± 1	6 ± 2	5 ± 2	2 ± 1	1 ± 1
	KYDSTHGRYAGE	221223331	17 ± 2	13 ± 4	3 ± 1	1 ± 1	0 ± 0
Increasing	PVGNPEGPEKPN	222212313	3 ± 0	5 ± 3	9 ± 1	11 ± 2	11 ± 1
	GNPIDGKGPID	222212313	2 ± 2	1 ± 1	2 ± 1	4 ± 2	3 ± 2
	QERDPANLPWGSSN	322212323	2 ± 1	4 ± 3	6 ± 2	11 ± 2	9 ± 2
	QERDPANLPWGSS	322212323	2 ± 1	1 ± 1	1 ± 1	7 ± 2	6 ± 3
Fluctuating	YFHEDDKF	123213331	0 ± 0	9 ± 2	7 ± 0	2 ± 0	1 ± 0
	SSKTNPKRDWF	223233232	1 ± 1	3 ± 1	2 ± 1	1 ± 1	2 ± 1
	GKKLEDHPKF	223233322	0 ± 0	7 ± 1	5 ± 2	2 ± 0	2 ± 1
Constant	IDAPGHRDF	122213322	9 ± 2	7 ± 1	9 ± 0	8 ± 3	7 ± 2
	EKNVPLYKH	123233222	13 ± 1	12 ± 4	10 ± 1	9 ± 2	8 ± 3
	YDSTHGRYAGE	221213331	12 ± 2	11 ± 3	11 ± 2	12 ± 2	11 ± 1
	PLVGGHEGAGV	231112212	23 ± 2	30 ± 3	23 ± 4	28 ± 2	21 ± 0
	PLVGGHEGAG	231112212	8 ± 1	10 ± 2	12 ± 1	13 ± 3	13 ± 1


*Means of 3 independent repetitions ± standard deviation.*

## Discussion

By using a mass spectrometry-based approach coupled with appropriate statistical tools, we were able to shed light on the peptide content of a yeast extract-based fermentation medium, but also to identify on a large scale distinct patterns of peptide abundance variations during the growth of *Streptococcus thermophilus*. The YE displayed a high peptide diversity with more than 4,000 distinct peptides identified. It possibly contains even more peptides, as the identification of some of them is still technically challenging (Guillot et al., 2016; Bingeman et al., 2017). It seems to be a general feature of YEs, as similar results have been obtained using another Nucell^®^ YE provided by Procelys (data not shown). The number of identified peptides was large enough with an appropriate physicochemical diversity to enable a robust analysis of peptide utilization by *S. thermophilus* N4L. By pooling peptides into physicochemical classes, we were able (i) to identify consistent kinetic profiles, and (ii) to compensate partially for the overall low relative abundance levels of individual peptides. As this grouping procedure was based on peptide physicochemical properties, which are known to be leading factors for their use by bacteria, the temporal evolutions observed in selected classes can reasonably be considered to mainly reflect peptide utilization dynamics of the strain N4L.

Four relevant kinetic profiles of peptide utilization have been observed: stagnation, decrease, increase and fluctuation of spectral counts over time. These patterns might come from two plausible origins: transport inside the cells, and/or peptide cleavage mediated by an extracellular hydrolytic activity. This last case is especially suggested by the presence of increasing profiles. Indeed, as the fermentation was performed in batch mode, the most likely explanation is that some peptides must have been gradually hydrolyzed by the strain into smaller fragments. These fragments can share the same barcodes as other peptides of the initial medium. Some of them are likely to be used by the strain, and do not accumulate in large amounts in the medium. Some others are not, and progressively accumulate in the external medium during the growth. This hypothesis is supported by the fact that increasing classes are constituted of numerous scarce peptides, many of which were only detected in the latter stages of fermentation. The cell-envelope located protease PrtS is the most plausible effector of this increase but the membrane-anchored protease HtrA could also play a role (Guillot et al., 2016). The hypothesis of a variation of some spectral counts due to cell lysis cannot be completely ruled out. However, considering the high number of intracellular peptidases in *S. thermophilus* and their overall large panel of specificities (Christensen et al., 1999; Hols et al., 2005; Savijoki et al., 2006), the hypothesis of a significant peptide cleavage by intracellular peptidases released during cell lysis is very unlikely. Otherwise, all classes of peptides would have been impacted, regardless of their biochemical properties. Therefore, the observed peptide dynamics can be explained as follows: (i) decrease: transport and/or cleavage of initially present peptides; (ii) increase: accumulation of cleavage products at a higher rate than their transport (if any transport); (iii) stagnation: neither transport/cleavage nor accumulation, or both at similar rates; (iv) fluctuating profile: combination of transport/cleavage and accumulation within the same physicochemical class at various changing rates over time, or artifactual noise (peptide identification variability).

In that respect, the presence of a large group of decreasing basic peptides is noteworthy, and it is sensible to assume that this decrease is predominantly the consequence of transport. As a first reason, the decrease depended on the global physicochemical property of the peptides, and not on their amino acid sequence. This observation does not argue in favor of hydrolysis by serine-proteases such as PrtS and HtrA, whose activity is known to be strongly dependent on the amino acid sequence flanking the cleavage site (Perona and Craik, 1995; Siezen and Leunissen, 1997). Then, the main conclusion of our study is that this decreasing profile is firstly linked to a systematic presence of a global positive net charge combined with a significantly shorter length and a higher proportion of hydrophobic residues. This description perfectly matches that of the previously mentioned study performed with a protease-negative strain on a small number of milk-derived peptides (Juille et al., 2005). Our work, by relying on a vastly higher number of peptides, not only consolidates these former results but also suggests a dominant role of a positive net charge for peptide transport. It is thus reasonable to assume that most, if not all, of these decreasing peptides were actually preferentially consumed by the strain and transported inside the cells.

This transport was mediated by the Ami system which is the only oligopeptide carrier identified in the strain. It has been demonstrated in *L. lactis* that the oligopeptide-binding protein (OppA) primarily determines the overall peptide specificity of its cognate transporter (Doeven et al., 2004). Similarly, the consumption of positively charged peptides by *S. thermophilus* N4L is likely to be essentially dictated by its own oligopeptide-binding proteins, namely AmiA1 and AmiA3. Moreover, it has been formerly established *in vivo* that peptide transport in both species displayed very similar specificities (Juillard et al., 1998; Juille et al., 2005). Indeed, it was shown that *L. lactis* also preferentially used hydrophobic basic peptides ranging between 600 and 1,100 Da, although this bacterium can accommodate surprisingly long peptides up to 35 residues (Doeven et al., 2005), which is even longer than the maximal size (24 residues) observed with *S. thermophilus* (Garault et al., 2002). The ability of *L. lactis* to carry various sizes of peptides and its preference for hydrophobic peptides containing branched-chain amino acids – in particular isoleucine – were explained later on thanks to the crystallization of OppA (Berntsson et al., 2011, 2009). However, an apparent discrepancy remains in the literature between *in vivo* studies and structural characterization concerning the role of peptide net charge in *L. lactis* OppA-based selection. If this factor was determined *in vivo* as a major feature for transport (Juillard et al., 1998), it has not been identified as coming into play regarding binding mechanisms in structural data. In that perspective, it is noteworthy that the crystal structure of unliganded *E. coli* OppA binding site had revealed a negatively charged surface responsible for the preferential binding of basic peptides (Klepsch et al., 2011). This finding was subsequently found to apply as well to *S. typhimurium* OppA. Therefore, further work is needed to elucidate the role of peptide charge both in *L. lactis* and *S. thermophilus*, as corroborating evidence seems to indicate that this factor may be a widespread requisite feature for peptide transport.

Milk is considered as the natural ecological niche of *S. thermophilus*. The main source of amino acids during growth of *S. thermophilus* in milk are caseins. Analysis of amino acid composition of ß- and k-caseins (the caseins mainly cleaved by PrtS) reports a high prevalence of branched-chain amino acids (22.5% of the total amino acids of the two proteins), suggesting an efficient correlation between this amino acid composition and the preferences of the Ami transport system underlined in the present study. However, the frequency of positively charged amino acids in the casein sequences is in the same range as that of negatively charged amino acids (9.5 and 9.3%, respectively). As the positive net charge of peptides exerts a key role for peptide transport, it not anymore possible to connect the composition of charged amino acids in caseins to the preferences of the Ami system. It therefore indicates that the specificity of casein cleavage by PrtS will determine the capability of released peptides to be used by *S. thermophilus* during growth in milk.

## Conclusion

In conclusion, the identification of complex mixtures of peptides by mass spectrometry, although still technically challenging, is progressively gaining attention (Bingeman et al., 2017) and is proving to be an excellent exploratory approach to unravel the peptide content of complex media but also to study the diverse oligopeptide utilization patterns of a bacterial species during its growth. Combined with complementary approaches, it opens avenues for further characterization and optimization of protein hydrolysate-based culture media and could also be used to deepen our knowledge of bacterial physiology.

## Author Contributions

LP performed the experimental study. LP and EH performed the MS analyses. LP and VJ wrote the manuscript. All authors contributed to conception and design of the study, to manuscript revision, and approved the submitted version.

## Conflict of Interest Statement

AS and IB were employed by company Lesaffre. MP was employed by Sacco S.r.l. The remaining authors declare that the research was conducted in the absence of any commercial or financial relationships that could be construed as a potential conflict of interest.

## Footnotes

1 www.yeastgenome.org
