# Supplementary material for: Insights Into the Complexity of Yeast Extract Peptides and Their Utilization by Streptococcus thermophilus
Source: Front Microbiol. 2019 Apr 30;10:906. doi: 10.3389/fmicb.2019.00906 (PMC6524704; doi:10.3389/fmicb.2019.00906)
Supplement: Supplementary file 1 [file Data_Sheet_1.pdf]

Insights into the complexity of yeast extract peptides and their utilization by *Streptococcus thermophilus*

Lucas Proust, Alain Sourabié, Martin Pedersen, Iris Besançon, Eloi Haudebourg, Véronique Monnet and Vincent Juillard

Supporting information

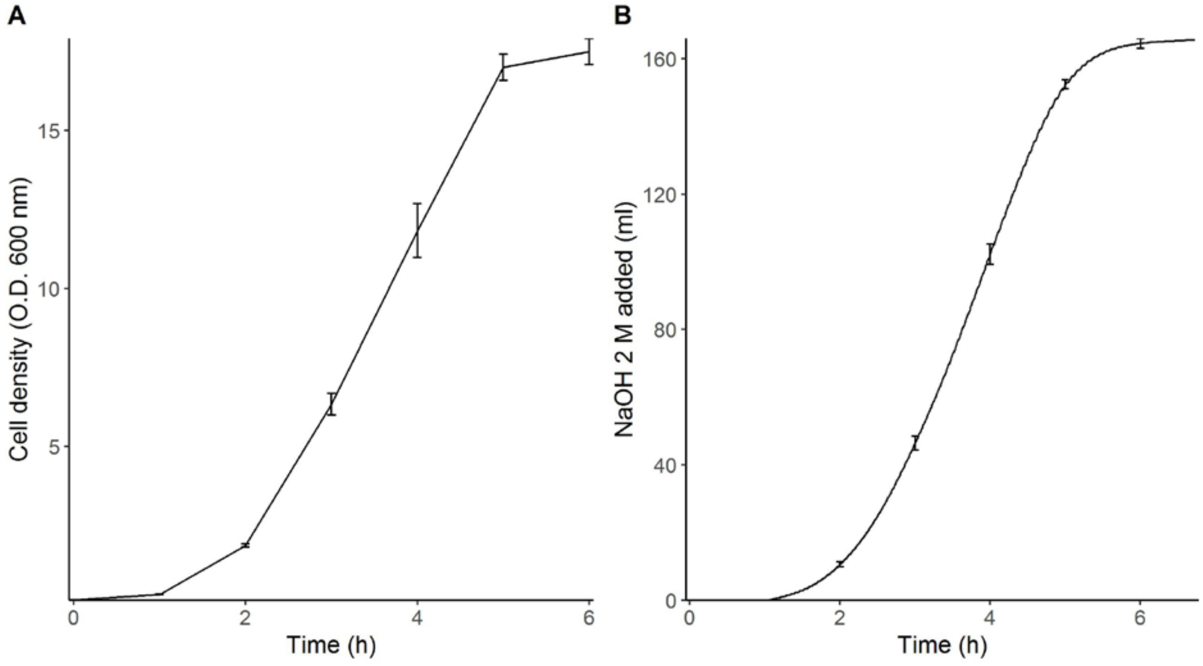

Figure S1. Growth of *S. thermophilus* in YE-based medium throughout fermentations performed at pH 6. (A) Bacterial biomass evaluated by optical density measured each hour at 600 nm. (B) Online monitoring of NaOH (2 M) addition to the fermentation medium as a consequence of pH regulation. The values correspond to the means of three independent experiments; errors bars represent the standard deviations.

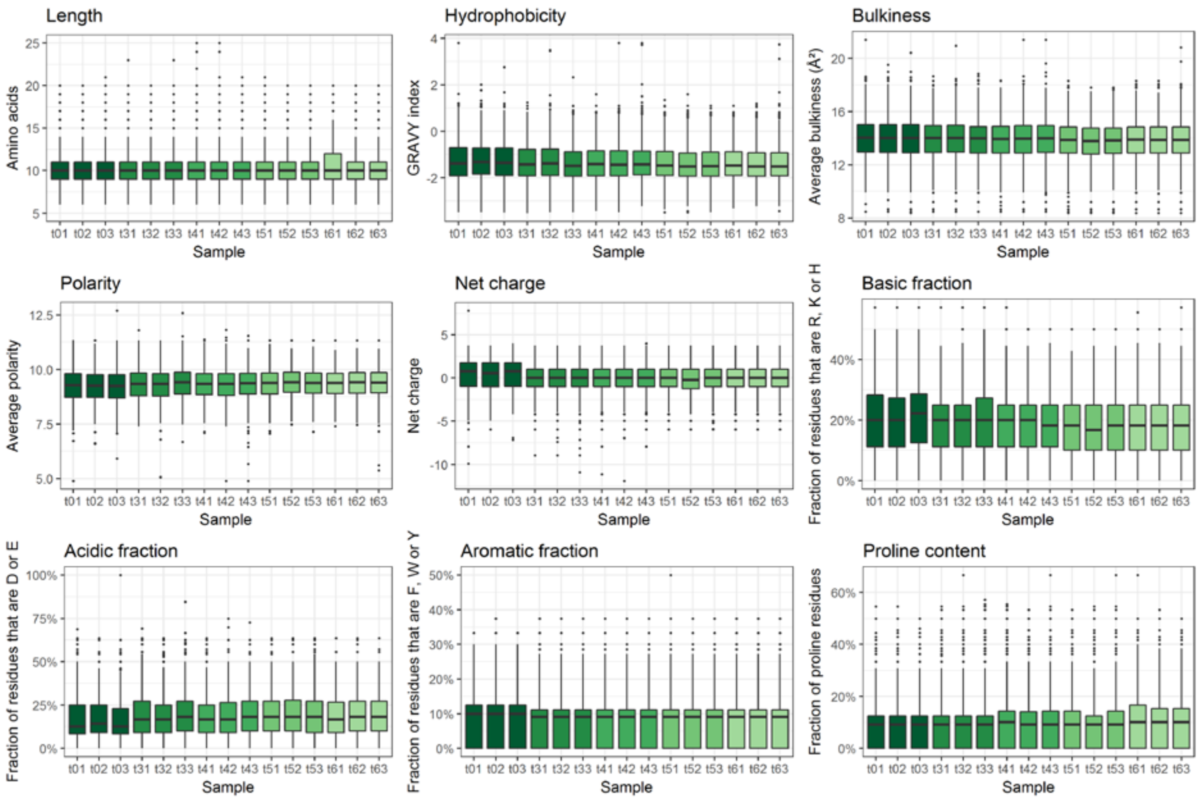

Figure S2: Physicochemical properties of the identified peptides in YE-based growth medium as a function of the growth course. Each box plot represents the distribution of the total spectra identified in one repetition, each shade of green one sampling time. From left to right: initial time (0 h), 3 h, 4 h 5 h and 6 h of bacterial growth. All repetitions are displayed as indicated in the x-axes (e.g. t01 corresponds to the first repetition at t = 0 h). The boxes display the range between the first and third quartile of each distribution, and the central bold lines their median value. Individual points are peptide spectra considered as outliers (more than 1.5 times the length of the box away from the box closest extremity).

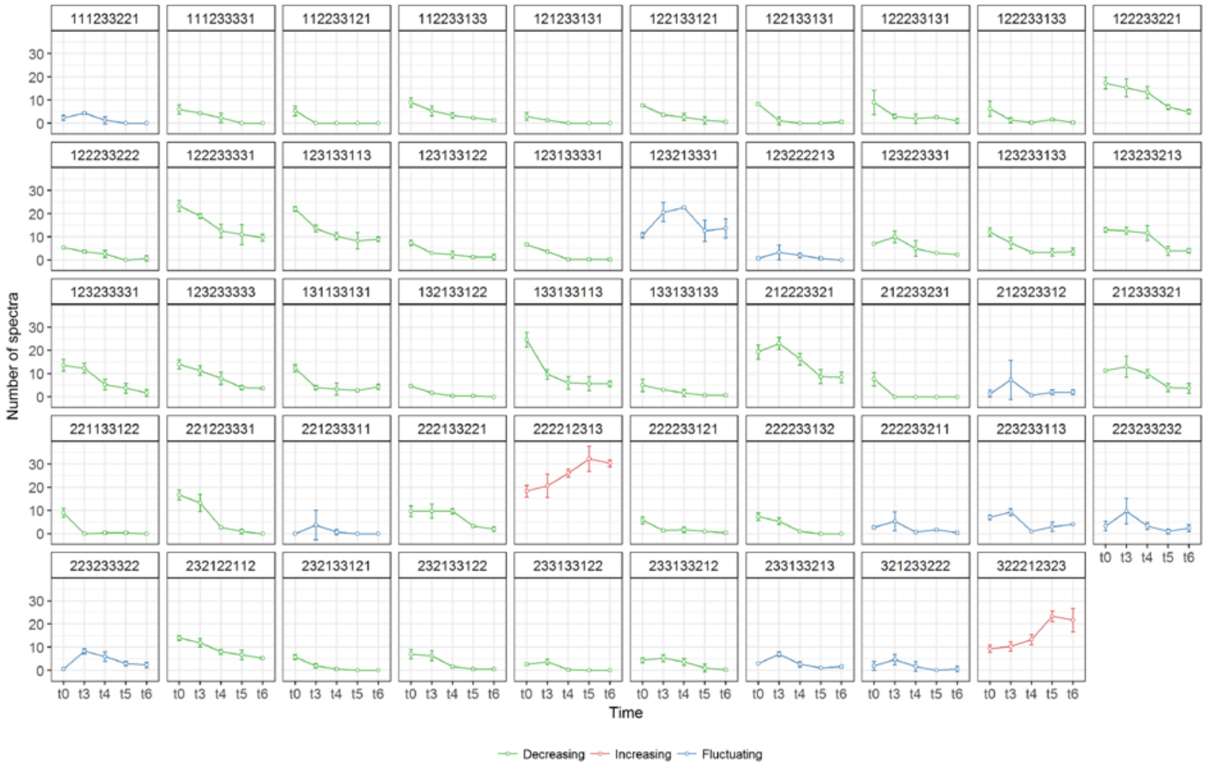

Figure S3: Classes of peptides whose population is significantly modified (adjusted p-val ≤ 0.01) during growth of *S. thermophilus*. The values correspond to the means of the spectra used to identify the peptides constituting the classes. The error bars represent the standard deviations. In green are classes showing a clear decrease over time, in red an increase over time, and in blue a class whose abundance evolution is fluctuating over time because of successive increase, decrease and stagnation.
